# Vertical and Horizontal Trophic Networks in the Aroid-Infesting Insect Community of Los Tuxtlas Biosphere Reserve, Mexico

**DOI:** 10.3390/insects10080252

**Published:** 2019-08-15

**Authors:** Guadalupe Amancio, Armando Aguirre-Jaimes, Vicente Hernández-Ortiz, Roger Guevara, Mauricio Quesada

**Affiliations:** 1 Red de Interacciones Multitróficas, Instituto de Ecología A.C., Xalapa, Veracruz 91073, Mexico; 2 Red de Biologia Evolutiva, Instituto de Ecología A.C., Xalapa, Veracruz 91073, Mexico; 3 Laboratorio Nacional de Análisis y Síntesis Ecológica, Escuela Nacional de Estudios Superiores Unidad Morelia, Universidad Nacional Autónoma de México, Morelia 58190 Michoacán, Mexico; 4 Instituto de Investigaciones en Ecosistemas y Sustentabilidad, Universidad Nacional Autónoma de México, Morelia 58190 Michoacán, Mexico

**Keywords:** phytophagy, trophic interactions, specialization, Diptera, Coleoptera, Hymenoptera, Lepidoptera

## Abstract

Insect-aroid interaction studies have focused largely on pollination systems; however, few report trophic interactions with other herbivores. This study features the endophagous insect community in reproductive aroid structures of a tropical rainforest of Mexico, and the shifting that occurs along an altitudinal gradient and among different hosts. In three sites of the Los Tuxtlas Biosphere Reserve in Mexico, we surveyed eight aroid species over a yearly cycle. The insects found were reared in the laboratory, quantified and identified. Data were analyzed through species interaction networks. We recorded 34 endophagous species from 21 families belonging to four insect orders. The community was highly specialized at both network and species levels. Along the altitudinal gradient, there was a reduction in richness and a high turnover of species, while the assemblage among hosts was also highly specific, with different dominant species. Our findings suggest that intrinsic plant factors could influence their occupation, and that the coexistence of distinct insect species in the assemblage could exert a direct or indirect influence on their ability to colonize such resources.

## 1. Introduction

A major goal of community ecology has been understanding the extent to which natural communities are structured by ecological processes, including competition for resources, predation or parasitism [1]. Herbivore trophic webs account for nearly 40% of global terrestrial biodiversity and are largely concentrated in tropical environments, playing a key role in the evolution of biodiversity, the assembly and dynamics of biotic communities and the functioning of ecosystems [2,3]. The mega-diversity of tropical herbivores is a result of several factors, including the greater presence of host plants and arthropods per plant species, along with higher host specificity, or increased species turnover [4]. Some authors [5,6] propose that tropical environments promote more specialized interactions, while the diversity of plant lineages, in turn, supports sets of comparatively highly specialized herbivores.

In tropical environments, the high fidelity of animals and plants to spatial or temporal habitat is understood as apparently minor differences in physical conditions, as compared with temperate habitats [7]. It supposes that animals and plants are evolutionarily adapted, or able to acclimate to temperatures that usually occur in their temporal and geographic habitat. Nevertheless, assuming that a host plant is optimal for the herbivore, it may be restricted to a narrow portion of the abiotic gradient due to interactions with the plant and its competitors. This enables the herbivore to adapt to the optimal abiotic gradient for the plant, becoming specialized both by the resource and the abiotic conditions [8].

Several hypotheses have been proposed to explain ecological gradients, including ones based on historical perturbation, environmental stability, habitat heterogeneity, productivity, speciation or interspecific interactions [9]. In this sense, elevation gradients display pronounced abiotic variations over extremely short geographic distances, therefore species at either low or high elevations may experience remarkably distinct abiotic shifts [10]. Differences in temperature are characteristics of altitudinal gradients, affecting ecological factors such as host plant quality, predation, parasitism and competition, since they can restrict the species distribution and choice of host plants [11]. Herbivorous insects tend to be ecologically specialized in the use of particular plant species, since the hosts are the site of most life activities, including feeding, oviposition and development of immature stages, pupation, even predator avoidance or reproduction [12,13]. It is widely accepted that plant-herbivore interactions concern both partners because plants exert selective pressure on herbivore features while the herbivores exert an influence on plant defense traits, promoting trends toward specialization and speciation [14,15]. Some ecological causes of reproductive isolation are influenced by the manner in which conspecific herbivore populations use divergent phenological hosts, the narrow synchronization between herbivore development and plant phenology, and the time of herbivore mating in different hosts [16]. Because of the multidimensional and multi-scale nature of niches, ecological specialization is one of the most multi-faceted concepts of ecology and, by definition, cannot be fully measured [17]. Several authors agree that the definition of ecological specialization depends largely upon approach of trophic levels, whether from individuals, species, populations to communities, and their relationships to spatial and temporal scales [8,14,18].

A meta-analytical assessment of interspecific interactions [19] provided overwhelming evidence that competition is indeed an important factor, affecting the performance and fitness of phytophagous insects. It has been argued that newly divergent specialist insects spend most of their life-history living on different hosts, reducing their likelihood of encountering each other when feeding or mating. Conversely, inter-specific competition between non-related insects exerts a strong adaptive competitiveness through a variety of mechanisms [20]. In this regard, some authors [21,22,23] have pointed out that, although intraspecific or paired interactions could be selective agents in a community, the indirect effects of multiple ecological interactions (e.g., exploitation competition, trophic cascades, apparent competition, indirect reciprocity and modification of interactions) should be incorporated into community-level phenomena in order to better understand and predict the dynamics of natural systems. Indirect interactions can thus be assessed by analyzing the trophic structure of insect communities, a task for which quantitative food networks are particularly suitable [1].

It is now widely accepted that analysis of complex networks is a useful tool for drawing predictive conclusions about biological communities in order to promote the persistence and stability of biodiversity [24,25]. In this way, antagonistic interaction networks contribute to the description of both multi-level and specific ecological processes, characterized by highly compartmentalized architecture and low connectivity implying lower nestedness than mutualistic networks [26,27,28].

The Araceae family possesses a high diversity in the tropical regions of the world, represented by 1,889 species in the Neotropical region [29], with nearly 109 species recorded in Mexico, and 55 in the state of Veracruz [30]. Aroids possess a typical inflorescence consisting of a spadix and a spathe, and the major differentiation among genera is the conversion from hermaphrodite to unisexual flowers, with the consequent separation into male and female sections. They are always protogynous and the female and male phases do not usually overlap, so obligatory cross-pollination seems to be the rule within the family [31]. The inflorescence is thermogenic during anthesis, which has proven to be an advantageous process for maximizing pollination and limiting hybridization [32]. During this procedure, they also produce scents, along with food resources such as stigmatic fluids, pollen, or sterile flowers to attract their pollinators [33,34,35,36,37].

However, such displays are also attractive to other insects for oviposition sites and breeding, although their role is uncertain since they are not necessarily implicated in the pollination process. Studies of insect-aroid trophic interactions are scarce in proportion to the diversity of this plant family. Florivory has been reported in *Peltandra virginica*, which is pollinated by the fly *Elachiptera formosa* Loew (Chloropidae) during oviposition within the inflorescence, in order to complete its life cycle [35]. Whereas floral predation by larvae of several richardiid fly species (Richardiidae: Diptera) has also been reported in *Gearum brasiliense* [38], *Dieffenbachia oerstedii* [39], *Taccarum ulei* [40], and some *Philodendron*, *Xanthosoma*, and *Rhodospatha* species [41]. Other surveys conducted in the Americas report highly diverse communities in *Xanthosoma* and *Syngonium*, involving up to nine visitor species of *Drosophila* [42], sixteen Dipteran species reared from *Symplocarpus foetidus* [43] and 15 species associated with three *Xanthosoma* species from central and South America, comprising pollinators and other Coleopteran, Dermapteran, Dipteran and Hemipteran taxa [44].

Since the aroids are a remarkable component of the hemiepiphytic vegetation and understory of the Los Tuxtlas Biosphere Reserve, and harbor a distinctive entomofauna inhabiting their inflorescences, we wonder how the aroid-infesting insect community differs in terms of altitude and specificity among host plants. The specific objectives were to: (1) describe the interactions of the endophagous insect community co-habiting within the infructescence of eight native species living in the rainforest of “Los Tuxtlas”, Mexico; (2) assess modifications in the assembly of species along an altitudinal gradient; and (3) characterize the shifts of the insect-community structure among host plant species.

## 2. Materials and Methods

### 2.1. Study Site

The Los Tuxtlas Biosphere Reserve (LTBR) is located in the southeastern part of Veracruz state, Mexico (18°05′–18°43′ N, 94°35′–95°25′ W). This region is the northernmost distributional limit of the evergreen tropical rainforest in the Americas, characterized by a complex topography with elevations from 0 to 1600 m asl, approximately [45,46]. Sampling was conducted at three sites of different elevations. The lowest site, “Biological Station of Tropical Biology Los Tuxtlas and surroundings” (BSLT), is found at 100–300 m asl characterized by climate subtype Am warm-humid with a summer rainy season and winter rainfall of between 5–10%, annual rainfall of 4001–5000 mm and an average temperature of 24.1–25 °C. The middle site “La Perla” is located at 500–700 m asl, with climate subtype Af (m) warm-humid, featuring rains throughout the year, winter precipitation of <18% and an average temperature of 23.1–24 °C with annual rainfall of between 4001–5000 mm. The highest site “Calería” is located at 1000–1200 m asl, with climate subtype (A)C (fm) semi-warm, with a temperature in the coldest month (January) of <18 °C and an average temperature of 22.1–23 °C [47,48].

Based on floristic studies [49,50], the natural vegetation at BSLT is a “tropical rainforest”, characterized by canopies of over 30 m in height, featuring elements such as *Ficus* spp., *Ceiba pentandra*, *Poulsenia armata*, *Nectandra ambigens*, *Brosimum alicastrum*, among others, while the understory is dominated by palms, especially *Astrocaryum mexicanum* and *Chamaedorea* species. At “La Perla”, the canopies do not exceed 20 m in height, and present a floral composition similar to that of the lower site (BSLT). The upper site of our study (Calería) is characterized by a transition zone, known as “Cloud Forest” (*sensu* Rzedowski [51]), with typical tree species such as *Liquidambar styraciflua*, *Alfaroa mexicana*, *Juglans olanchana*, *Ulmus mexicana*, among others. In the region of Los Tuxtlas, aroids are an important element of the native vegetation, represented by 34 species of nine genera, distributed from 0 to 1600 m asl, at the top of the San Martin volcano. The best-represented genera in terms of the highest number of species are *Philodendron*, *Anthurium*, *Monstera* and *Syngonium* [52].

### 2.2. Collection and Breeding of Biological Samples

From August 2015 to March 2017, five sampling events were conducted along transects of approximately 1000 m at each altitudinal level, searching for mature infructescences from eight aroid species as available. All samples were labeled prior to transportation to the laboratory for further inspection. Each sample was individually placed in a plastic breeding chamber and maintained under laboratory conditions (24 °C ± 4; RH = 70% ± 10), and diurnal cycles of 12 h [39]. Mature infructescences were dissected for inspection, and larvae or pupae were placed in breeding chambers until the adult emergence, recording the abundance of species feeding inside. Adult individuals were preserved in ethanol 75%, and some dry mounted on pins for identification. Reference insect specimens will be deposited in the IEXA entomological collections (INECOL Xalapa, Veracruz, Mexico). Sampled plants were identified by photographs taken in situ, and later compared with specimens deposited in the herbarium of the BSLT-UNAM.

Observations of feeding behavior were made during breeding of the immature phases in the laboratory. Following taxonomic identification, and supported by our observations and the literature [53], a classification by trophic guilds was conducted: (1) phytophagous: insect-feeding only from fresh tissues (including spathe, spadix, flowers or fruits); (2) phytophagous-saprophagous: insect-feeding from fresh and mature tissues; (3) saprophagous: insect-feeding only from decomposing tissues; and (4) mycetophagous: insect-feeding presumably from fungi associated with decomposing tissues.

### 2.3. Statistical Analyses

The assemblage of aroid-infesting insect communities, including site-host comparisons, were assessed through trophic interaction networks, measuring the overall specialization index for the community (H2’), the degree of specific specialization (d′), which measures host selectivity on the part of the herbivores, and the high-level niche overlap (NO) [54,55]. Modularity (*Q*) was calculated using the function compute-Modules in the package bipartite, which applies the DIRTLPAwb+ algorithm [56] based on the quantitative matrix of interactions. Significance tests for the selected settings were performed by running 100 randomizations of the null Patefield model and the resulting values were then used to calculate the Z-score (Z ≥ 2, high significance), which measures the deviation from one value exceeding the average of 100 randomized networks [57]. Network metrics were calculated with the package bipartite v. 2.11 [58].

In order to assess the specific interaction network among plants and insects, the “*hub*” species (insect herbivores) and “*authorities*” (plants hosts) were determined with Kleinberg’s centrality scores of the adjacency matrix of the network using abundance data standardized by the Log10(x) + 1 function [59], with the functions *hub-score* and *authority-score* of the package igraph [60]. Functional guild proportions among hosts were assessed by means of a G-test of independence with the Williams correction [61], using the package DescTools [62].

Nonparametric estimators were used to determine the total number of species throughout the samples. We constructed species accumulation curves for the observed and expected number of species, using Jackknife 1 based on abundance, because of its efficiency and reduced dependence on sample size [63]. Beta diversity across elevations (at both community and host levels) was assessed through the abundance-based dissimilarity Bray-Curtis index (βBC), using the “beta.multi.abun” and “beta.pair.abun” functions of the Betapart package [64]. Dissimilarity is equally affected by (i) balanced changes in species abundances between sites (B = C, i.e., the abundance of some species declines from one site to the other at the same magnitude that the abundance of other species increases); and (ii) abundance gradients (B > C = 0, i.e., the abundance of all species declines or increases equally from one site to the other) [65].

Since the absolute abundance rates per host ignored the number of infructescences infested, a frequency/abundance index (IFA) was estimated for each insect species, which linked the number of infructescences occupied with the number of insects detected in the sample.

$$IFA=rf_{i}\times ra_{i}\times 100$$

where:

*IFA* = Index of frequency/abundance of species *i* found in the host plant
$rf_{i}$ = relative frequency of species *i* in the host plant (percentage of infructescence infested).
$ra_{i}$ = relative abundance of species *i* in the host plant (percentage of individuals recovered in the sample). Unless stated otherwise, all analyses were carried out with the statistical environment “R” v. 3.5.1 [66].

## 3. Results

### 3.1. Aroid-Insect Interaction Network

We surveyed a total of 250 infructescences from eight aroid species: *Dieffenbachia oerstedii*, *Philodendron inaequilaterum*, *P. seguine*, *P. radiatum*, *P. sagittifolium*, *P. tripartitum*, *Rhodospatha wendlandii* and *Xanthosoma robustum*. Their average fresh weight ranged from 15.74 ± 1.12 g for *P. inaequilaterum* to 211.66 ± 6.88 g in *P. radiatum*. Forty-nine percent of the infructescences (123) were collected at the lowest elevation (BSLT), while 37.6% (94) and 13.2% (33) were obtained at the middle (La Perla) and highest (Calería) sites, respectively. Of the eight plant species sampled, *D. oerstedii*, *P. tripartitum*, and *P. sagittifolium* accounted for 70% of total infructescences, while *P. inaequilaterum* accounted for just nine infructescences (Table 1).

Eight aroid species harbored a total of 16,120 insect individuals. Sampling effort, based on species accumulation curves, detected a total of 34 species, which corresponded to 78.5% of the 43.3 species expected with Jackknife 1. Meanwhile, the sampling coverage estimated for each altitudinal level varied between 84.03% at low-level, 77.7% at mid-level, and 67.6% at high-level, respectively, representing a good level of sampling completeness. Insect species belonged to four orders, and only four of these accounted for 74.5% of all specimens: *Aprostocetus* sp. (Hymenoptera, Eulophidae) (36.1%), *Symplecta* sp. (Diptera, Tipulidae) (20.0%), Nitidulidae-1 (Coleoptera) (11.0%) and *Beebeomyia tuxtlaensis* (Diptera, Richardiidae) (7.5%). The order Diptera comprised of 25 species from 15 families, among which, the most important were Richardiidae, Stratiomyidae, Tipulidae, Scatopsidae and Drosophilidae, which together accounted for 12 species with nearly 90% of the dipteran abundance in the entire sample. The order Coleoptera consisted of seven species, including four from the family Nitidulidae, whereas the families Curculionidae, Scarabaeidae and Ptiliidae were each represented by a single species. *Aprostocetus* sp. (Hymenoptera: Eulophidae) was a gall-forming wasp found in high numbers in fruits of *Philodendron radiatum*, including a single Lepidopteran species recorded by four larvae (Pyralidae) feeding as spadix-borer of this plant. All of these findings are virtually the first record of host plants in the Neotropical region. The number of species recovered per host was highly variable, ranging from two in *R. wendlandii* up to 18 in *P. tripartitum*, although only a few species represented more than 90% of the abundance for each host (Table 2).

**Figure 1:**
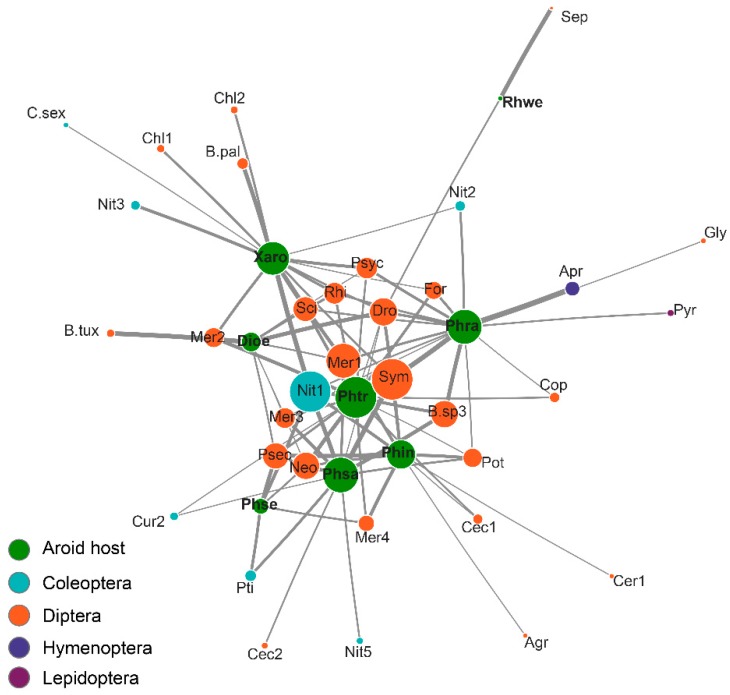
Insect-aroid interaction network in infructescences of eight species in the Los Tuxtlas Biosphere Reserve, Mexico. Circle size is proportional to the number of links. Line thickness is proportional to the number of individuals. The species with the greatest number of interactions are located centrally.

The overall interaction network exhibited high modularity (Q = 0.53, Z = 294.18, *p* < 0.001), a high degree of specialization (H2’ = 0.78, *p* < 0.001) and a low niche overlap among herbivore species (NO = 0.21, Z = −6, *p* < 0.001). Insect-plant interactions within this community proved to be highly specialized for 91% of insect species. The most specialized taxa (d’ = 0.49–0.99) belonged to the order Diptera in the families Richardiidae (3), Scatopsidae (1), Stratiomyidae (2) and Lonchaeidae (1), along with Eulophidae (Hymenoptera), and one species of Nitidulidae (Coleoptera). Meanwhile, all eight aroid species sampled also exhibited high specialization levels (d’ = 0.48–0.99). The Kleinberg hub analysis revealed that *P. sagittifolium*, *P. tripartitum* and *P. radiatum*, as well as *X. robustum* (authority score 1.0–0.39), displayed the largest degree of connectivity. The insect species with the highest connectivity indices were Nitidulidae-1 and Stratiomyidae (3), besides of Tipulidae, Lonchaeidae, Richardiidae, Sciaridae, Scatopsidae and Drosophilidae, with a single species each (hub-score 1.0–0.44) (Figure 1, Table 3).

### 3.2. Vertical Distribution

The vertical stratification analysis of the community revealed that the interaction networks present a high modularity pattern and degree of specialization (Q = 0.43 ± 0.05, H2’ = 0.73 ± 0.09 SE). Aroid species’ richness decreased with elevation, as seven species were found at lowest level, five in the middle and only three at the highest elevation. Similarly, the greatest richness and abundance values of insects were recorded at the bottom level (25 species, 72.8% of specimens), compared to the middle (22 species, 21.6% of specimens), and upper (14 species, 5.6%) elevations.

**Figure 2:**
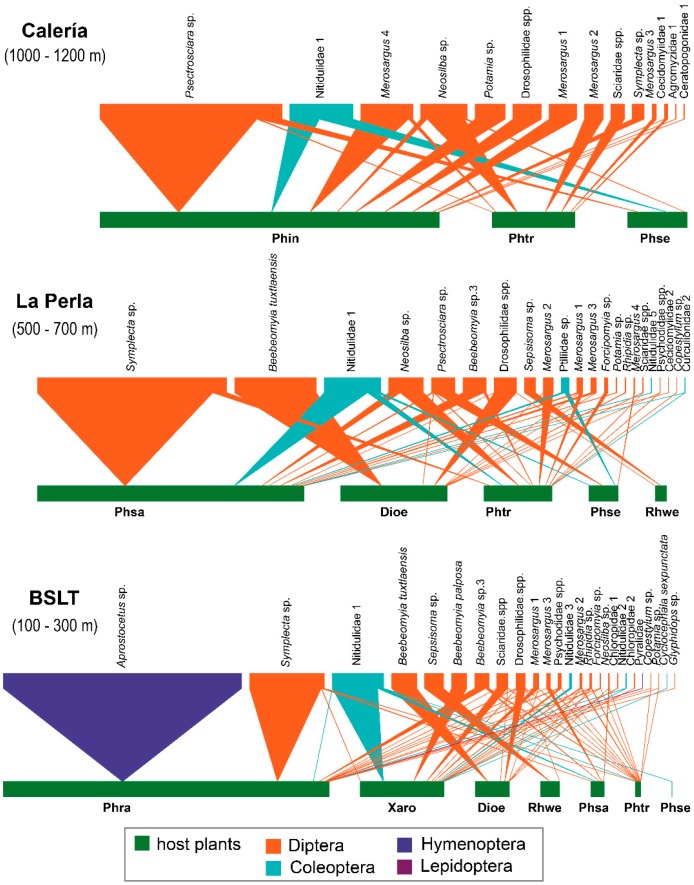
Insect-aroid interaction networks at three altitudinal levels: high site Calería, med site La Perla, and low site BSLT in Los Tuxtlas Biosphere Reserve, Mexico. The bottom bars depict plant species and top bars depict insects. Full names for each taxon are provided in Table 1.

Overall, the most significant hub-species experienced a progressive reduction in abundance with elevation. The species with the highest connectivity indices, such as Nitidulidae-1, showed a notable decrease of up to ten times in value from the lowest to the highest elevation, while *Merosargus*-1 (Stratiomyidae) presented twice as many individuals at the low level compared to the higher elevations; *Symplecta* sp. (Tipulidae) presented similar abundances at the lower and middle levels, declining notably almost to disappearance at the highest site. Only two aroid species occurred along the entire gradient: *P. tripartitum*, which presented a decreasing number of interactions from the lowest to the highest site with 14, 12 and 6 species, respectively. In contrast, *P. seguine* exhibited a higher number of interactions at the middle and high elevations (4, 3), with only one present at the lowest site. The genus *Philodendron* featured the largest number of insects per altitudinal level; *P. radiatum*, *P. sagittifolium* and *P. inaequilaterum*, each containing one dominant phytophagous species from the lowest to the highest levels; the gall-forming wasp *Aprostocetus* sp., the tipulid fly *Symplecta* sp. and the scatopsid fly *Psectrosciara* sp., respectively (Figure 2).

**Figure 3:**
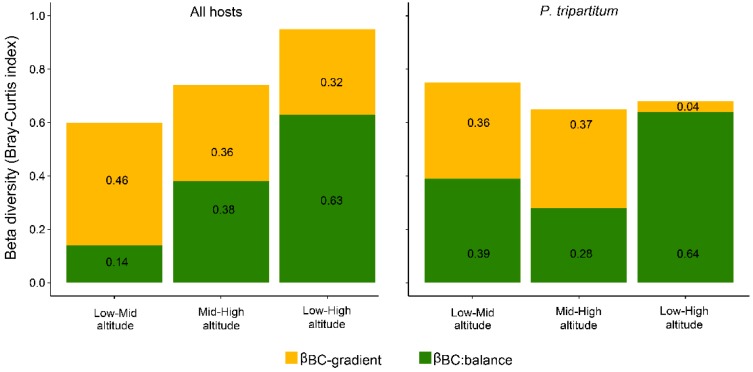
Comparisons of Bray-Curtis dissimilarity indices showing Beta diversity among the study sites of the whole insect community (left side), and of *P. tripartitum* (right side). Upper bars show βBC-gradient (abundance gradient) and lower bars show βBC-balance (balanced variation in abundance) (sensu Baselga 2013, 2017).

Variation in richness and abundance of herbivore insects along every level produced a high beta diversity for the entire community (βBC = 0.77). The most distant locations (BSLT vs. Calería) included 30 herbivores, with only nine shared species (βBC:balance = 0.63; βBC:gradient = 0.32), producing a loss of richness and significant shifts in abundance. Conversely, the low and intermediate altitudinal levels (BSLT and La Perla, respectively) together accounted for 31 species, 16 of which were common with little divergence found in abundance (βBC:balance = 0.14; βBC:gradient = 0.46) (Figure 3A).

In populations of *P.* *tripartitum* as the unique host distributed across the altitudinal range, an assemblage was also reported with significant modifications either in terms of richness or abundance (βBC = 0.73). Herbivore diversity decreased gradually from 14 species in BSLT, to 12 in La Perla and only 7 in Calería, with a notable replacement of species between extremes (BSLT vs Calería), a substantial reduction in shared species and high dissimilarities (βBC:balance = 0.64, βBC:gradient = 0.04). Meanwhile, comparisons at the assemblage of adjacent levels, BSLT vs. La Perla, revealed greater similarities (βBC:balance = 0.39; βBC:gradient = 0.36) (Figure 3B).

### 3.3. Horizontal Distribution among Host Plants

The network of trophic interactions of the entire community produced a highly specialized and modular system (H2’ = 0.56 ± 0.05, Q = 0.33 ± 0.06 SE), with extremely low niche overlap values (NO = 0.15 ± 0.03). These specialization levels were also reported for each host plant metric, implying a high preference for specific aroid plants. While the richness and abundance of insect assemblages were highly variable among hosts, there was a range of two interactions for *R. wendlandii* to 18 interactions observed for *P. tripartitum* (Table 4).

**Figure 4:**
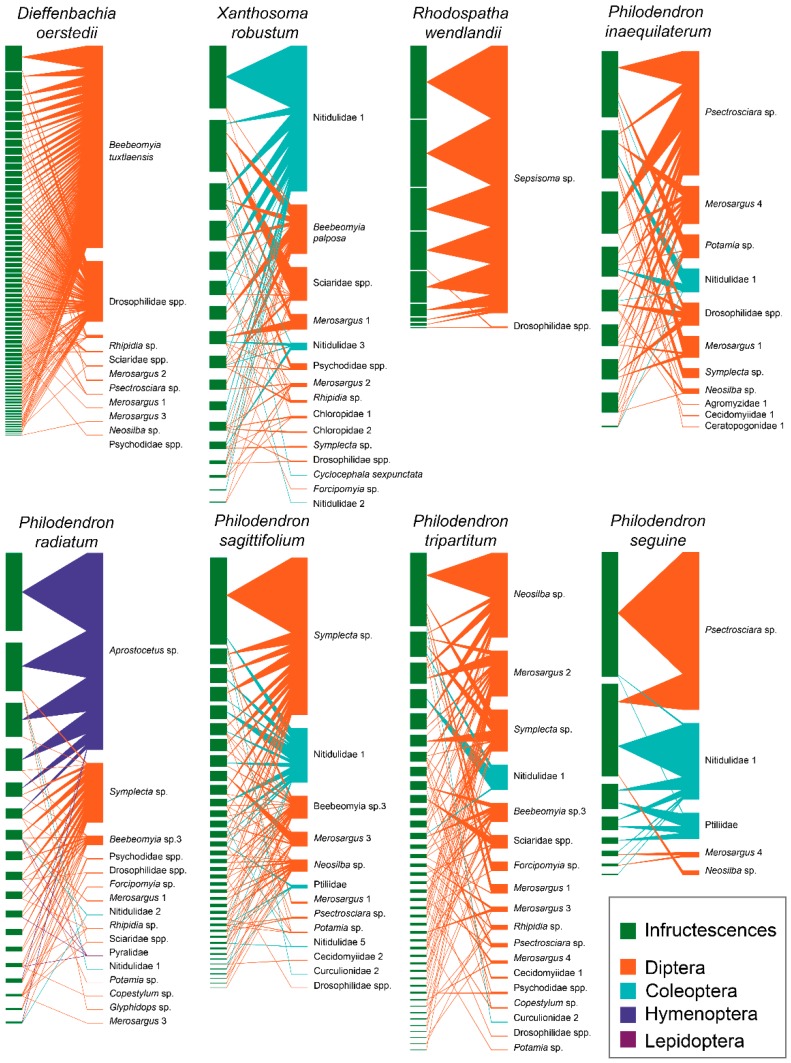
Insect-aroid interaction networks in eight plant species shown as individual infructescences on the left (green squares), and insect species on the right. The width of the bars denotes the number of individuals.

As stated above, individual interaction networks enabled the identification of some degree of specialization, either at generic or specific levels. For instance, the richardiid fly *B. tuxtlaensis* was only found in *D. oerstedii*; *Sepsisoma* sp. was exclusive to *R. wendlandii*; *B. palposa* was only encountered in *X. robustum*, while *Beebeomyia* sp. 3 was reared from three *Philodendron* species. Similarly, the wasp *Aprostocetus* sp. was only associated with *P. radiatum*, and the tipulid fly *Symplecta* sp. was primarily associated with three *Philodendron* hosts. Conversely, some dipteran species of the Stratiomyidae and Drosophilidae families, as well as the beetle Nitidulidae-1, were encountered in the majority of the hosts, albeit with significant changes in abundance (Figure 4).

Based on the IFA scores calculated, 11 species ranked as dominant herbivores for the entire community (1 Coleopteran, 9 Dipterans and 1 Hymenopteran). The highest values were reported for *B. tuxtlaensis* (70.01) and *Sepsisoma* sp. (88.4), followed by *Psectrosciara* sp. (46.2), *Symplecta* sp. (26.1, 18.3) and *Aprostocetus* sp. (32.5); in addition to the beetle Nitidulidae-sp1 (42.7, 24.9, 6.9), which was highlighted by high scores obtained from three different hosts (Table 5).

**Figure 5:**
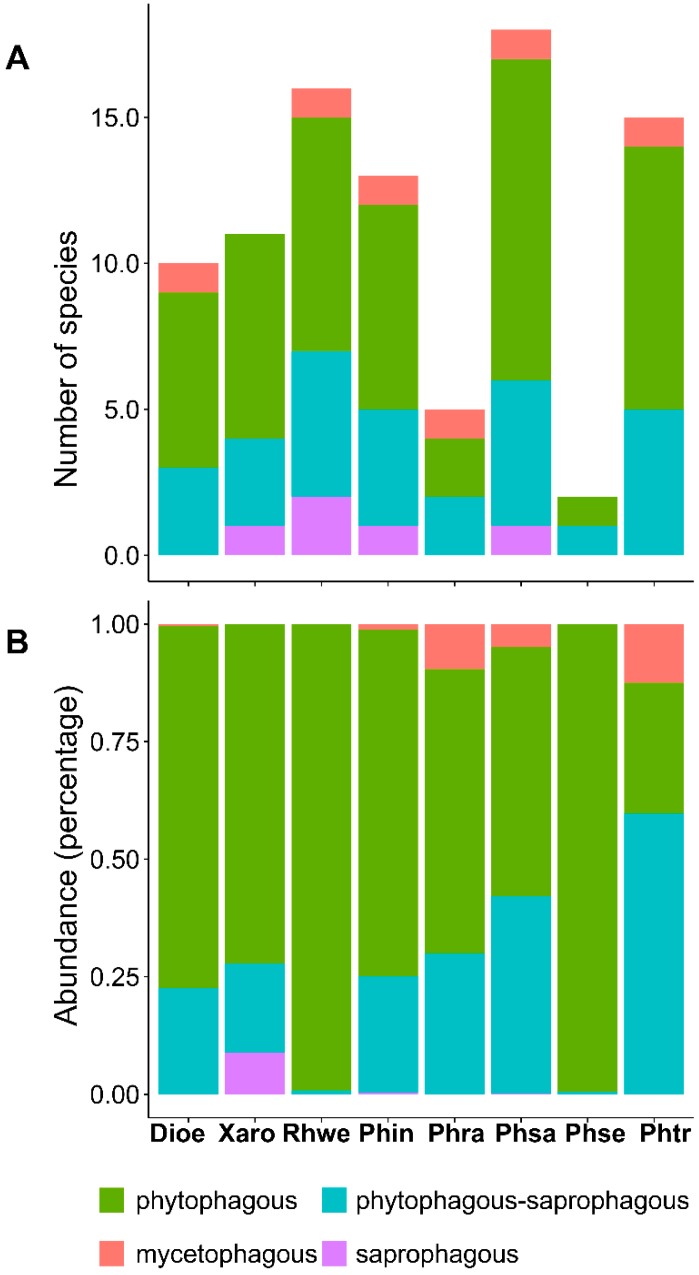
Abundance and richness of functional groups encountered in the community of endophagous insects per host examined. (**A**) Number of species per host; (**B**) Proportion of total abundance by guild. The full names for each taxon are provided in Table 1.

In terms of larval habits, we defined a priori four functional groups within the system. The phytophagous guild presented the highest species richness (64.7%, 22 spp.), followed by a transitional saprophagous-phytophagous guild (23.5%, 8 spp.), and two other guilds, mycetophagous (1 spp.) and saprophagous (1 spp.), which were barely represented. All four groups were detected in *P. radiatum*, *P. sagittifolium* and *P. tripartitum*, whereas *D. oerstedii*, *P. seguine* and *X. robustum* did not contain any saprophagous species, and only two groups were detected for *R. wendlandii*. Based on the rates of abundance, the phytophagous guild was best represented (80.3%); however, in terms of frequency/abundance, the functional groups differed significantly among the plant species (G = (21) 6651.66, *p* < 0.001) (Figure 5).

## 4. Discussion

No comprehensive studies are available describing the trophic interactions of the herbivore-aroid community in the tropics of the Americas. In the Nearctic plant *Symplocarpus foetidus*, 16 Dipteran species (Ceratopogonidae, Chloropidae, Drosophilidae, Ephydridae and Psychodidae) were recorded, along with coleopteran, psocopteran and collembolan species [43]. As a result of this study, we characterized the richness of insect diversity feeding on aroid reproductive tissues. Thirty-four insect species were identified, the larvae of which are dependent on the aroid infructescence for survival and development, thus providing a niche that can explain part of the herbivore diversity in tropical environments.

Structural parameters of herbivore-plant interaction networks are typically compartmentalized with few generalist species, but are also spatially and temporally dynamic, and can be influenced by seasonality, habitat complexity, and food resource availability [25,26,27,28,67,68]. The overall network of trophic interactions exhibited a high degree of specialization and modularity in our system, both among hosts and along the elevation gradient. As a result, its architecture was highly compartmentalized, with low connectivity among species and niche overlap. On the horizontal level, herbivore distribution among hosts exhibited some shared species, although abundances differed greatly (e.g., Nitidulidae-1, Drosophilidae spp, *Neosilba* sp, *Merosargus*-1, *Symplecta* sp), evidencing that each species performed better in a particular host, rarely in two, while in other hosts they were extremely rare, except in the case of Nitidulidae-1, which became a hub-species of the entire system, since it was detected in six plants, with significant abundances in at least five of them.

It has been reported that climatic factors significantly influence patterns of insect diversity and species turnover [69,70,71]. In this sense, it is known that notable climatic variations occur among studied sites, since average annual temperatures decrease by 3 °C, while rainfall increases by up to 2000 mm between elevational extremes (BSLT vs. Calería) [48]. Herbivore complexity showed a proportional reduction with elevation, in terms of the numbers of both phytophagous species and individuals, as well as significant variations in species composition and dominance. The insect community exhibited the highest richness at the lowest elevation, gradually decreasing to nearly one-third of that value at the upper elevation (Calería), whereas only nine out of all of the species were recorded at all levels, including mixed dominance ranges [Nitidulidae-1, Drosophilidae spp, Stratiomyidae (3), Lonchaeidae (1), Muscidae (1), Sciaridae spp., Tipulidae (1)]. Replacement of dominant species at different levels may be related to resource availability, since not all hosts were found along the entire transect. However, some evidence suggests that herbivores possess inherent limitations in terms of the use of a single resource at higher elevations. For instance, the species *P. tripartitum*, which was present at all three altitudinal levels, presented significant shifts in both composition and species richness, in addition to changes in the dominant species; while *Beebeomyia* sp. 3 (Richardiidae) was most abundant in the BSLT, it was *Neosilba* sp. (Lonchaeidae) at the middle and upper elevations (La Perla and Calería).

Reproductive traits in Araceae are intimately linked to pollination strategies [72,73]. Therefore, we assumed that herbivore assemblages within each infructescence would also be influenced by its particular morphological features. However, some contrasting traits among hosts, such as infructescence biomass, did not have any effect on herbivore abundance or infestation rates, as seen in flies of the family Richardiidae [41]. The lowest endophagous richness was found in *R. wendlandii* (two species), since it possesses a deciduous spathe that is lost after anthesis, and thus does not form a chamber, while the ripening fruits remain exposed along the spadix. On the other hand, assemblages of 5–18 herbivores were recorded in *Philodendron*, *Xanthosoma* and *Dieffenbachia*, which present a perennial spathe that closes after anthesis, providing a chamber for fruit protection, and also a suitable niche for the growth of phytophages that lay their eggs inside [39,43,74,75,76].

The traditional theory of competition states that competitive interactions are symmetrical, requiring spatial and temporal co-occurrence, and their intensity increases with increasing density, phylogenetic similarity and overlapping niches of competitor species. However, a meta-analysis of interspecific competition among herbivorous insects [19] revealed that indirect interactions provide the most evidence of competition in herbivore interactions, suggesting that interspecific effects are highly asymmetrical and occur at relatively low densities, or between divergent taxa feeding at different times, and on different plant tissues. The discovery of a complex community of herbivores cohabiting within, and collectively depending on aroid infructescences for survival, suggests that indirect interspecific competition may be occurring in our system.

The relationship between oviposition preference by females and offspring performance is a critical issue in the evolutionary ecology of insect-plant interactions [77]. It means that offspring survive better in preferred plant types and that females lay more eggs on plant types that will favor larval performance, which is known as the preference-performance hypothesis [78]. In this context, we noted some highly specialized taxa in different parts of the infructescence meaning that, even inside this structure, a niche partition exists that permits exploitation by different species. For instance, we found larvae mainly consuming the spathe (e.g., *Symplecta* sp., Tipulidae); fruit-feeding larvae (e.g., Stratiomyidae, Lonchaeidae); fruit-gall forming wasp larvae (*Aprostocetus* sp., Eulophidae) and a pyralid larva (Lepidoptera) feeding as a spadix borer.

Some phytophages of aroids possess mechanisms to avoid competition between conspecific females, such as the use of dissuasive pheromones to prevent oviposition in a single structure [39].

In addition, other authors have described chemical compounds such as calcium oxalates, present during inflorescence maturation and considered a deterrent of phytophagy [31,79,80,81,82]. Conceptually, plant-induced defenses increase the potential competition between two species, even if they occur at different times in the host or are exploiting different parts of the structure [7]. Variations in concentrations of calcium oxalates occur between different host species or tissues of the same plant [80,83]. However, there is no evidence of such variations in different habitats or with elevations. Remarkable variations in the abundance of specific herbivores within a single host may be explained by differences in the concentrations and degradation times of secondary metabolites of the infructescence, in addition to different levels of specialization expected by the herbivores living there. These aspects merit further investigation.

From the perspective of functional groups, the boundaries between phytophagy, saprophagy or mycetophagy are unclear. Indeed, the insect species reported here presumably oviposited before, or most likely during anthesis, and some are strictly phytophagous, because their larvae feed on living tissues, which exhibited evident damage from feeding (e.g., Richardiidae, Tipulidae, Scatopsidae, Nitidulidae). However, other species were classified as phytophagous-saprophagous, since they remained inside the infructescence from hatching, until the ripening fruits began their degradation process (e.g., Stratiomyidae, Lonchaeidae, Syrphidae). Given the complexity of multi-species interactions, interference in the feeding process could be expected and thus some species may restrict colonization by others, in addition to the inherent limitations of non-specialist phytophages. For example, the inflorescence of *D. oerstedii* is primarily colonized by *B. tuxtlaensis* (mostly on the female section), the larvae of which trigger a decomposition process in the tissues, which are then exploited by the drosophilid larvae [39].

The aroids largely depend on insects for sexual reproduction and thus display several resources for attracting pollinators, such as pollen, starch-rich staminodes, and sweet stigmatic secretions [84,85]. It has been argued that the beetles Scarabaeidae (Cyclocephalinae), Nitidulidae and Staphilinidae are typically the main aroid pollinators, as well as some Euglossinae or Trigoninae (Hymenoptera) and a few Dipteran families Drosophilidae, Sciaridae, Psychodidae and Chloropidae [72,73,86,87,88], and references herein). Considering the high abundance and specificity levels found in some insect families (e.g., Nitidulidae, Drosophilidae, Scatopsidae, Psychodidae, Sciaridae and Tipulidae), it is speculated that some of them may not only be attracted to and feed upon these structures, but could also be implicated in the reproductive processes of their hosts. Again, this aspect requires further investigation.

## 5. Conclusions

We studied a system of insect-aroid trophic interactions in Los Tuxtlas Biosphere Reserve, Mexico. We found a high species diversity of insects that were dependent on the inflorescences to fulfill their life cycle, forming a complex community of 34 species from 21 families belonging to four insect orders. Nearly all of them are recorded for the first time feeding on plants of the family Araceae, highlighting the importance of the reproductive structures of these plants as a hot-spot reservoir for tropical rainforest biodiversity. The assemblage of the entire herbivore community was characterized by highly specialized trophic interactions, mainly represented by monophagous or stenophagous species exhibiting low levels of connectivity between modules. From a diversity perspective, a gradual reduction was observed with increasing elevation, both in terms of the number of plants and insects, and also a significant replacement of the phytophages, which could be related to the decreased temperatures and increased rainfall from the lower to the upper studied sites. Horizontally, a high degree of specialization is maintained in the system, consisting of distinct dominant species with a low similarity in the assemblages, since few species were shared among hosts. Our results suggest that the indirect interactions within aroid infructescences, along with host morphology and secondary metabolites, could restrict their colonization and be responsible for the structuring of the trophic interactions present in those plant communities.

## Figures and Tables

**Table 1:** Infructescences from eight aroid species collected at three different elevations (shown in parentheses) in Los Tuxtlas Biosphere Reserve, Mexico.

Aroid Species	BSLT (1000–1200 m)	La Perla (500–700 m)	Calería (100–300 m)	Total	Percentage of Total Sample
*Dieffenbachia oerstedii* (Dioe)	44	33	-	77	30.8%
*Xanthosoma robustum* (Xaro)	18	-	-	18	7.2%
*Rhodospata wendlandii* (Rhwe)	17	2	-	19	7.6%
*Philodendron inaequilaterum* (Phin)	-	-	9	9	3.6%
*Philodendron radiatum* (Phra)	18	-	-	18	7.2%
*Philodendron sagittifolium* (Phsa)	7	38	-	45	18.0%
*Philodendron seguine* (Phse)	1	6	4	11	4.4%
*Philodendron tripartitum* (Phtr)	18	15	20	53	21.2%
Totals	123	94	33	250	
Percentage of the sample	49.20%	37.60%	13.20%		

**Table 2:** List of the insects reared from the eight aroid plants showing the percentage of abundance per host. The highest abundance species per host plant are shown in bold. Trophic guilds are as follows: P = phytophagous, S = saprophagous, M = mycophagous, P-S = phytophagous-saprophagous.

Family	Species Name	Species Code	Trophic Guild	*Dioe*	*Phin*	*Phra*	*Phsa*	*Phse*	*Phtr*	*Rhwe*	*Xaro*	Total Specimens
Curculionidae	Curculionidae-2	Cur2	P	-	-	-	0.04	-	0.13	-	-	2
Nitidulidae	Nitidulidae-1	Nit1	P-S	-	8.68	0.04	20.15	28.48	9.33	-	54.14	1781
	Nitidulidae-2	Nit2	P-S	-	-	0.14	-	-	-	-	0.05	12
	Nitidulidae-3	Nit3	P-S	-	-	-	-	-	-	-	2.79	57
	Nitidulidae-5	Nit5	P-S	-	-	-	0.18	-	-	-	-	4
Ptiliidae	Ptiliidae	Pti	M	-	-	-	1.15	9.6	-	-	-	57
Scarabaeidae	*Cyclocephala sexpunctata*	C.sex	P	-	-	-	-	-	-	-	0.05	1
Agromyzidae	Agromyzidae-1	Agr	P	-	0.16	-	-	-	-	-	-	1
Cecidomyiidae	Cecidomyiidae-1	Cec1	P	-	0.16	-	-	-	0.65	-	-	6
	Cecidomyiidae-2	Cec2	P	-	-	-	0.13	-	-	-	-	3
Ceratopogonidae	Ceratopogonidae-1	Cer1	P	-	0.16	-	-	-	-	-	-	1
	*Forcipomyia* sp	For	P	-	-	0.23	-	-	3.5	-	0.05	46
Chloropidae	Chloropidae-1	Chl1	P	-	-	-	-	-	-	-	0.69	14
	Chloropidae-2	Chl2	P	-	-	-	-	-	-	-	0.49	10
Drosophilidae	Drosophilidae-spp.	Dro	P-S	22.53	8.36	0.24	0.04	-	0.13	0.55	0.34	445
Lonchaeidae	*Neosilba* sp.	Neo	P-S	0.06	1.89	-	4.39	1.55	31.35	-	-	359
Muscidae	*Potamia* sp.	Pot	S	-	8.83	0.03	0.36	-	0.13	-	-	67
Neriidae	*Glyphidops* sp.	Gly	S	-	-	0.01	-	-	-	-	-	1
Psychodidae	Psychodidae-spp.	Psyc	P-S	0.06	-	0.36	-	-	0.65	-	2.35	83
Richardiidae	*Beebeomyia palposa*	B.pal	P	-	-	-	-	-	-	-	18.23	372
	*Beebeomyia* sp3	B.sp3	P	-	-	3.41	8.43	-	6.87	-	-	514
	*Beebeomyia tuxtlaensis*	B.tux	P	75.16	-	-	-	-	-	-	-	1204
	*Sepsisoma* sp.	Sep	P	-	-	-	-	-	-	99.45	-	543
Scatopsidae	*Psectrosciara* sp.	Psec	P	0.25	46.21	-	0.58	58.51	1.55	-	-	511
Sciaridae	Sciaridae-spp	Sci	M	0.37	-	0.06	-	-	4.92	-	12.49	304
Stratiomyidae	*Merosargus*-1	Mer1	P	0.19	8.04	0.16	0.71	-	3.37	-	5.68	225
	*Merosargus*-2	Mer2	P	0.31	-	-	-	-	16.84	-	1.27	162
	*Merosargus*-3	Mer3	P	0.06	-	0.01	5.37	-	1.81	-	-	137
	*Merosargus*-4	Mer4	P	-	14.04	-	-	1.86	1.04	-	-	102
Syrphidae	*Copestylum* sp.	Cop	P-S	-	-	0.01	-	-	0.52	-	-	5
Tipulidae	*Rhipidia* sp.	Rhi	P	1	-	0.09	-	-	1.68	-	0.93	55
	*Symplecta* sp.	Sym	P	-	3.47	21.99	58.46	-	15.54	-	0.44	3216
Eulophidae	*Aprostocetus* sp.	Apr	P	-	-	73.17	-	-	-	-	-	5816
Pyralidae	Pyralidae	Pyr	P	-	-	0.05	-	-	-	-	-	4
Insect species	34			10	11	16	13	5	18	2	15	16,120

**Table 3:** Descriptors of network trophic interactions of insects feeding in aroid infructescences at three different elevations in Los Tuxtlas Biosphere Reserve, Mexico.

Network Metrics	BSLT	La Perla	Calería	Full Network
Aroid species	7	5	3	8
Insect species	25	22	14	34
Modularity (*Q*)	* 0.465	* 0.504	* 0.325	* 0.523
Specialization (*H_2_’*)	* 0.875	* 0.745	* 0.571	* 0.781
Insect niche overlap	0.31	0.31	0.51	0.21

* denotes significant values for modularity and specialization (*p* < 0.05).

**Table 4:** Descriptors of the network trophic interactions of the insect community feeding in eight aroid plants in Los Tuxtlas Biosphere Reserve, Mexico.

Network Metrics	*Dioe*	*Phra*	*Phin*	*Phsa*	*Phse*	*Phtr*	*Rhwe*	*Xaro*
Infructescences (n)	73	18	9	38	8	41	14	17
Insect species	10	16	11	13	5	18	2	15
Modularity (*Q*)	* 0.234	* 0.356	* 0.308	* 0.391	* 0.387	* 0.544	* 0.009	* 0.428
Specialization (*H2’*)	* 0.391	* 0.744	* 0.344	* 0.595	* 0.68	* 0.618	* 0.577	* 0.556
Insect niche overlap	0.03	0.19	0.29	0.08	0.18	0.08	0.25	0.14

* denotes significant values for modularity and specialization (*p* < 0.05).

**Table 5:** Index of frequency-abundance (IFA) showing the insect species with highest values per host plant.

Host Plants	Number of Sampled Infructescences	Insect Species	Relative Frequency	Relative Abundance	IFA
*D. oerstedii*	73	*B. tuxtlaensis*	0.932	0.752	70.01
		Drosophilidae spp.	0.562	0.225	12.66
*X. robustum*	19	Nitidulidae-1	0.789	0.541	42.72
		*B. palposa*	0.737	0.182	13.45
*P. sagittifolium*	38	*Symplecta* sp.	0.447	0.585	26.15
		Nitidulidae-1	0.342	0.202	6.89
*P. inaequilaterum*	9	*Psectrosciara* sp.	1.00	0.462	46.21
		*Merosargus*-4	0.667	0.14	9.36
*P. seguine*	8	Nitidulidae-1	0.875	0.285	24.92
		*Psectrosciara* sp.	0.25	0.585	14.63
*P. radiatum*	18	*Aprostocetus* sp.	0.444	0.732	32.52
		*Symplecta* sp.	0.833	0.22	18.33
*P. tripartitum*	41	*Merosargus*-2	0.537	0.168	9.04
		*Neosilba* sp.	0.22	0.313	6.88
*R. wendlandii*	9	*Sepsisoma* sp.	0.889	0.995	88.4
